# 

**Published:** 1925-01

**Authors:** 


					CONTENTS.
Page
residential Address: Postgraduate Medical Education.
By
J- Odery Symes, M.D., D.P.H.
-uuuauun. JL>y
^PPend
. 'citis as a Cause of Intestinal Obstruction.
By Richard
w
akren, M.D., M.Ch.Oxon., F.R.C.S
IVlUriAKU
23
A Q
Ofnparative Review of Spinal and Peripheral Nerve Injuries.
By Wilfrid Adams, M.S., F.R.C.S.
nci vc 111j ui ico.
32
ev'ews of Books
43
^d'torial
Notes
53
Me
et'ngs of Societies
55
Obi
'tuary:
Lemuel Matthews Griffiths, M.R.C.S., L.R.C.P.
61
Robert H. Norgate, M.R.C.S., L.R.C.P.
65
Cecil Clarke, M.D., B.S., M.R.C.P. Lond.
66
I ?.
u,orary of the Bristol Medico-Chirurgical Society
Lnfs.l ..
69
L?Cal
Medical Notes
7i

				

